# Toxic Effects of Docosahexaenoic Acid Treatment in the Rat Liver BRL-3A Cell

**DOI:** 10.3390/toxics9050112

**Published:** 2021-05-20

**Authors:** Wenli Luo, Li Li, Weina Xu, Jing Zhang, Jianxiong Xu

**Affiliations:** Shanghai Key Laboratory of Veterinary Biotechnology, School of Agriculture and Biology, Shanghai Jiao Tong University, Shanghai 200240, China; maple_rowe@sjtu.edu.cn (W.L.); lili_1996@sjtu.edu.cn (L.L.); xuweina@sjtu.edu.cn (W.X.); zhangjing224@sjtu.edu.cn (J.Z.)

**Keywords:** apoptosis, BRL-3A, cytokine, DHA, MAPK, NF-κB

## Abstract

The cytotoxicity of docosahexaenoic acid (DHA) on normal cells is still unclear. This study investigated the effects of DHA on the cytotoxicity and possible mechanism in the BRL-3A cell. The cultured rat liver BRL-3A cell line was treated with 50, 100 and 200 μM DHA for 24 h. The cell viability was increased in the 50 and 100 μM DHA treatments, but decreased in the 200 μM DHA treatment. The 50, 100 and 200 μM DHA treatments increased the proportion of the apoptotic cells, the levels of lactate dehydrogenase (LDH), alkaline phosphatase (AKP) and IL-6 in the supernatant, and the ratio of the phosphonated p38MAPK to the p38MAPK (p-p38/p38) protein in the cells. The expression of TGF beta-activated kinase 1 (TAK1), nuclear transcription factor-κB p65 (NF-κB p65) and the inhibitor of NF-κB alpha (IκBα) mRNA, and the ratio of the phosphonated IκBα (p-IκBα) to IκBα protein were increased in the 200 μM DHA treatment, while the ratio of phosphonated extracellular regulated protein kinases (p-ERK) to ERK protein was decreased in the 200 μM DHA treatment. These results indicate that DHA-treated (50, 100 and 200 μM) BRL-3A cells for 24 h promotes cell apoptosis and inflammatory response, and the p38 MAPK, ERK and NF-κB signal pathways were involved in mediating the apoptosis and inflammatory response.

## 1. Introduction

Docosahexaenoic acid (DHA) is a kind of n-3 long-chain polyunsaturated fatty acid (n-3 LC-PUFA) and is widely recognized to prevent metabolic disorders, such as coronary heart disease, atherosclerosis and gestational diabetes mellitus, through reducing the inflammatory response and oxidative stress [1,2,3,4]. The supplementation of DHA in immune cells and adipocytes can reduce the inflammatory response in immune cells under inflammatory stimulation [1]. DHA has cytotoxicity to various types of cancer cells (e.g., lung cancer cell). Thus, the supplementation of DHA in daily diet is a common nutritional method to prevent the occurrence and development of metabolic disorders or chronic diseases. The market value of the EPA and DHA ingredient exceeded US $2.3 billion in 2019, and is expected to grow at over 7.2% CAGR between 2020 and 2026 according to a report of Global Market Insights, Inc. [5].

Although the consumption of DHA has continued to increase, the report about the cytotoxicity of DHA on normal cells is limited and the safety of DHA is still unclear [1,6]. Notably, there is no report about the upper limit of tolerable intake of DHA [7]. Zajdel et.al (2010) reported that DHA pre-treatment increased the sensitivity of non-cancer cells to drug toxicity. The cell activity of rat pancreatic β cells (RINm5f) decreased after 100 μM DHA treatment for 24 h [8]. The large amount of DHA intake has adverse effects on immune function, antioxidant capacity and lipid or glucose metabolism [7]. Our previous animal experiment showed that the maternal diet with fish oil (enriched DHA) increased the levels of IL-1β and IL-6 in the plasma of piglets and caused liver injury pre-/post-LPS challenge [9].

NF-κB and MAPK (including p38, ERK and JNK) signal pathways regulate the immune response, stimulating pro-inflammatory cytokine production to promote inflammation [10]. DHA supplementation can not only reduce the inflammatory response by inhibiting the phosphorylation of IκB α and JNK [1], but can also cause cytotoxicity by inhibiting the activity of ERK phosphorylation in the 24-h exposure experiment. As mentioned above, we hypothesize that the DHA might have toxic effects on normal cells, which might be mediated by the NF-κB and MAPK signal pathways. In this study, BRL-3A cells from normal rat liver were used to study. The objective of this study was to investigate the effect of different concentrations of DHA on the cytotoxicity and the possible mechanism. It will provide reference for further study on the mechanism of DHA in regulating liver inflammation.

## 2. Materials and Methods

*Cell Culture.* The normal rat liver BRL-3A cell line was purchased from the Y-J biological Co., Ltd. (Shanghai, China). BRL-3A cell line was maintained in complete medium, including 89% Dulbecco’s modified Eagle’s medium with 4.5 g/L D-glucose (item no.11995065, Gibco, Thermo Fisher, MA, USA), 10% fetal bovine serum (item no.10091148, Gibco, Thermo Fisher, MA, USA) and 1% penicillin/streptomycin (item no.15140122, Gibco, Thermo Fisher, MA, USA). Cells were incubated in a humidified incubator (37 °C, 5% CO_2_).

*Cell Treated with DHA*. DHA was purchased from Cayman Chemical Company (item no.90310, Ann Arbor, MI, USA). We made the DHA working solution as 2 mM and diluted with culture medium gradually. The required concentrations of DHA were freshly diluted with the culture medium before each experiment. The BRL-3A cells were seeded in a standard plate at the density of 2 × 10^5^ cells/mL. The medium was removed after culturing for 6 h and replaced by the same culture medium with the required concentrations of DHA.

*Measurement of Cell Viability.* The cells were maintained in the complete medium with the required concentrations of DHA (0, 50, 100, 150, 200, 250 and 300 μM) for 24 h. The cell viability of each well was determined by using CCK-8 assay kit (no. C0039, Beyotime Biotechnology, Shanghai, China). The CCK-8 regent in the assay kit was added into the wells of the control and treatment groups. The values of absorbance were measured at 450 nm by using a microplate reader (Synergy 2, BioTek, Winooski, VT, USA). The cell viability of the 0μM DHA treatment was set as 100% and the cell viability of other DHA treatments were calculated as (A450 treatment group)/(A450 control group) × 100%.

*LDH determination.* The cells were divided into groups as follows: the control group, DHA-treated groups at concentrations of 50, 100, 150, 200, 250 and 300 μM for 24 h. The level of LDH was determined by using LDH assay kit (no. C0016, Beyotime Biotechnology, Shanghai, China).

*Determination of Apoptotic Rates.* The cells were divided into groups as follows: the control group, DHA-treated groups at concentrations of 50, 100 and 200 μM for 24 h. The PBS washed cells twice after treatment. Cells were collected and centrifuged at 500× *g* for 5 min. The Cellometer Mini counted the number of cells. The cell density was adjusted to 5 × 10^4^. Cells were stained with fluorescein isothiocyanate (annexin V-FITC) and propidium iodide (PI). The proportion of apoptotic cells was detected by flow cytometry. The fluorescence of apoptotic cells was observed by fluorescence microscope (Nikon, ECLIPSE Ti-U).

Determination of the activities of aspartate aminotransferase (AST), alanine aminotransferase (ALT) and alkaline phosphatase (AKP) in supernatant. The cells were divided into groups as in the description above. The supernatant was collected from each well by centrifuging for 5 min at 500× *g* after treatment. The activities of AST, ALT and AKP were determined by kits (item no. C010-2, item no. C009-2, and item no. A059-2, respectively, Jiancheng Bioengineering Institute, Nanjing, China).

*Analysis of cytokines.* The cells were divided into groups as in the description above. The supernatant was collected after being DHA-treated for 24 h from each well by centrifuging for 5 min at 500× *g* after treatment. The levels of IL-1β, IL-6 and IL-10 in the supernatant were determined by using rat-specific ELISA kits (Jiancheng Bioengineering Institute, Nanjing, China). 

*RNA Extraction and qPCR Analysis.* The cells were divided into groups as follows: the control group, DHA-treated groups at concentrations of 50, 100 and 200 μM for 24 h. Cells were harvested after treatment. Total RNA were extracted and analyzed as described previously [11]. Primers for all target genes were referred to in the previous report (Table 1). The internal control gene was β-actin and each sample was replicated three times.

*Western Blotting Analysis.* BRL-3A cells were seeded in cell culture dishes (100 mm × 20 mm, 430167, Corning Inc., New York, NY, USA) and each group was treated as above. Cells were lysed on ice in RIPA buffer with 1 × cocktail protease inhibitor for 50 min and collected supernatants after centrifuging for 25 min at 12,000× *g*. The protein contents of each sample were detected by BCA protein assay kit according to the manufacturer’s instructions (P0010, Beyotime Biotechnology, Shanghai, China). The primary antibodies p38 (1:2000, sc-535, Santa Cruz, Dallas, TX, USA), p-p38 (1:200, sc-7973, Santa Cruz, Dallas, TX, USA), ERK1/2 (1:1000, number 9102, Cell Signaling Technology, Beverly, Danvers, MA, USA), p-ERK1/2 (1:2000, number 4370, Cell Signaling Technology, Beverly, Danvers, MA, USA), JNK (1:200, sc-571, Santa Cruz, Dallas, TX, USA), p-JNK (1:500, orb10951, Biorbyt Ltd., Cambridge, UK), IκBα (1:1000, number 4814, Cell Signaling Technology, Beverly, Danvers, MA, USA), and p-IκBα (1:1000, number 9246, Cell Signaling Technology, Beverly, Danvers, MA, USA) were used to determine the relative protein expression levels according to our previously described by Luo et al. [16].

*Statistical Analyses.* All data distributed normally by Shapiro–Wilk test and were analyzed by using the procedure of one-way ANOVA (IBM SPSS Statistics 20, Armonk, NY, USA). The differences among the treatment were analyzed by using one-way ANOVA multiple comparisons with LSD post-hoc test. Data were presented as means with standard error (SEM). A *p* value less than 0.05 indicated statistical difference.

## 3. Results

### 3.1. Effects of DHA Treated for 24 h in BRL-3A on the Cell Viability and the Release of LDH

**Figure 1:**
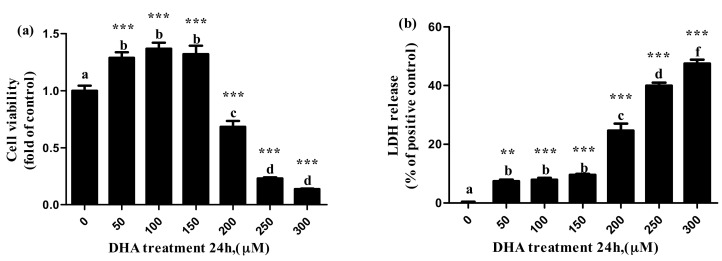
The effect of different concentrations of DHA-treated BRL-3A for 24 h on the cell viability (**a**) and lactate dehydrogenase release (**b**) (*n* = 5). Values are means with SEM, the SEM was presented with vertical bars; means with different letters are significantly different from one another (*p* < 0.05) as determined by variance analysis followed by multiple comparisons with LSD post-hoc test; ** statistically significant differences vs. control (*p* < 0.01); *** statistically significant differences vs. control (*p* < 0.001).

Compared with the control, the cell viability increased significantly with the increase in DHA from 50 μM to 150 μM (129 ± 4.8% at 50 μm, 137 ± 5.2% at 100 μm and 132 ± 7.2% at 150 μM), but the cell viability decreased significantly with the increase in DHA from 200 μM to 300 μM (68.4 ± 5.2% at 200 μM, 23.2 ± 0.8% at 250 μM and 13.8 ± 0.3% at 300 μM) (Figure 1a).

The release of LDH significantly increased with the increase in DHA from 50 μM to 150 μM, while the release of LDH significantly increased parallel to the increase in the DHA from 200 μM to 300 μM (Figure 1b).

### 3.2. Effects of DHA Treated for 24 h in BRL-3A on Lipid Profile

**Figure 2:**
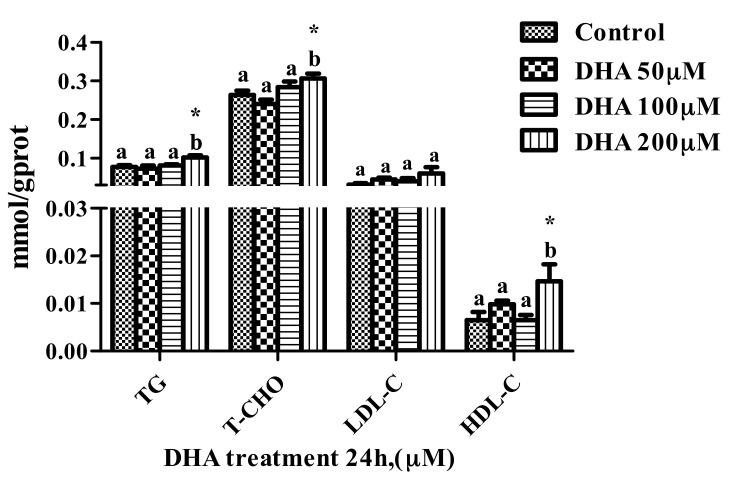
The effect of different concentrations of DHA-treated BRL-3A for 24 h on the lipid profile (*n* = 3). Values are means with SEM, the SEM was presented with vertical bars; means with different letters are significantly different from one another (*p* < 0.05) as determined by variance analysis followed by multiple comparisons with LSD post-hoc test; * statistically significant differences vs. control (*p* < 0.05).

The levels of T-CHO and HDL-C in the BRL-3A cells from the 200 μM DHA treatment were higher than those from the control and other treatment groups (*p* < 0.05) (Figure 2).

### 3.3. Effects of DHA Treated for 24 h in BRL-3A on Cell Apoptosis

**Figure 3:**
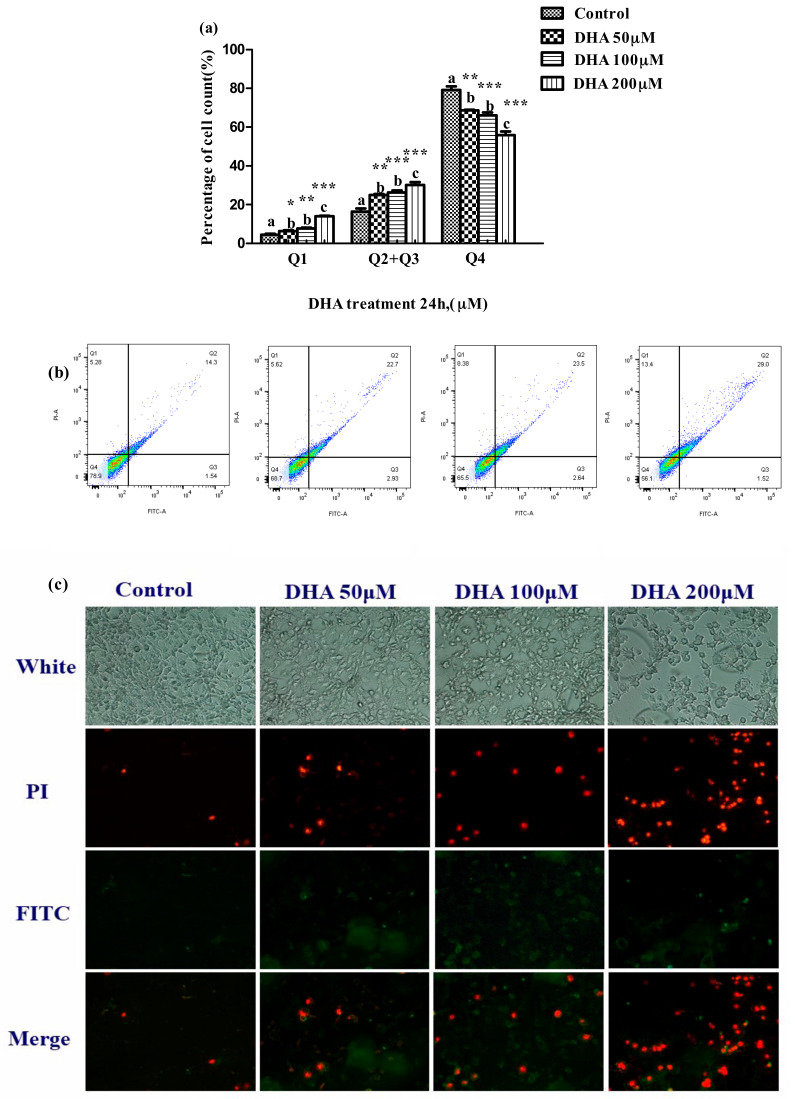
Effect of different concentrations DHA-treated BRL-3A for 24 h on the cell apoptosis. BRL-3A cells were stained with annexin V-FITC and PI after treatment with different concentration DHA, (**a**) the proportion of apoptotic cells was determined by flow cytometry (*n* = 3); values are means with SEM, the SEM was presented with vertical bars; means with different letters are significantly different from one another (*p* < 0.05) as determined by variance analysis followed by multiple comparisons with LSD post-hoc test; * statistically significant differences vs. control (*p* < 0.05); ** statistically significant differences vs. control (*p* < 0.01); *** statistically significant differences vs. control (*p* < 0.001); (**b**) the fluorescence of apoptotic cells was observed by fluorescence microscope (*n* = 3); (**c**) cells were treated as described in Section 2 and analyzed by fluorescence microscope.

The proportion of apoptotic cells was significantly increased in the 50, 100 and 200 μM DHA treatments in comparison to those in the control (*p* < 0.05) (Figure 3a).

### 3.4. Effects of DHA Treated for 24 h in BRL-3A on the Release of Liver Function Related Enzymes

**Figure 4:**
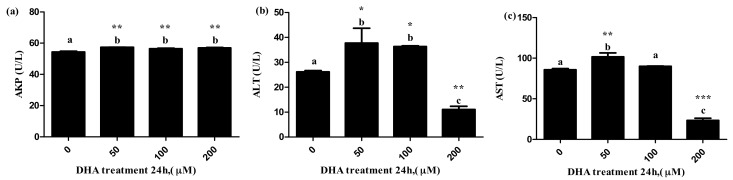
The effects of DHA-treated BRL-3A for 24 h on the activity of AKP (**a**), ALT (**b**) and AST (**c**) in the supernatants (*n* = 3). Values are means with SEM, the SEM was presented by vertical bars (*n* = 3); means with different letters are significantly different from one another (*p* < 0.05) as determined by variance analysis followed by multiple comparisons with LSD post-hoc test; * statistically significant differences vs. control (*p* < 0.05); ** statistically significant differences vs. control (*p* < 0.01); *** statistically significant differences vs. control (*p* < 0.001).

The 50 μM DHA treatment significantly increased the levels of ALT, AST and AKP in the supernatant, and the 100 μM DHA treatment significantly increased the levels of ALT and AKP in the supernatant (Figure 4a–c, respectively). The 200 μM DHA treatment significantly increased the level of AKP in the supernatant, but decreased the levels of AST and ALT in the supernatant (Figure 4c).

### 3.5. Effects of DHA Treated for 24 h in BRL-3A on the Release of Cytokines

**Figure 5:**
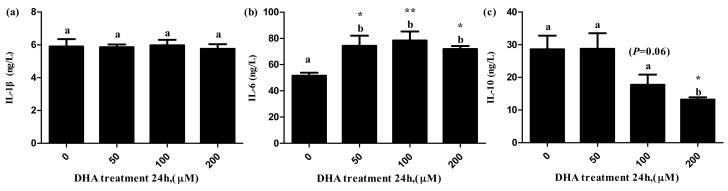
The effects of DHA-treated BRL-3A for 24 h on the contents of IL-1β (**a**), IL-6 (**b**) and IL-10 (**c**) in the supernatants (*n* = 3). Values are means with SEM, the SEM was presented by vertical bars (*n* = 3); means with different letters are significantly different from one another (*p* < 0.05) as determined by variance analysis followed by multiple comparisons with LSD post-hoc test; * statistically significant differences vs. control (*p* < 0.05); ** statistically significant differences vs. control (*p* < 0.01).

The level of IL-6 in the supernatant significantly increased in the 50, 100 and 200 μM DHA treatments (Figure 5b), but the level of IL-10 in the supernatant significantly decreased in the 200 μM DHA treatment (Figure 5c). There was significant tendency on the level of IL-10 in the supernatant of the 100 μM DHA treatment in comparison to the control (*p* = 0.06).

### 3.6. Effects of DHA Treated for 24 h in BRL-3A on MAPK and NF-κB Pathway

**Figure 6:**
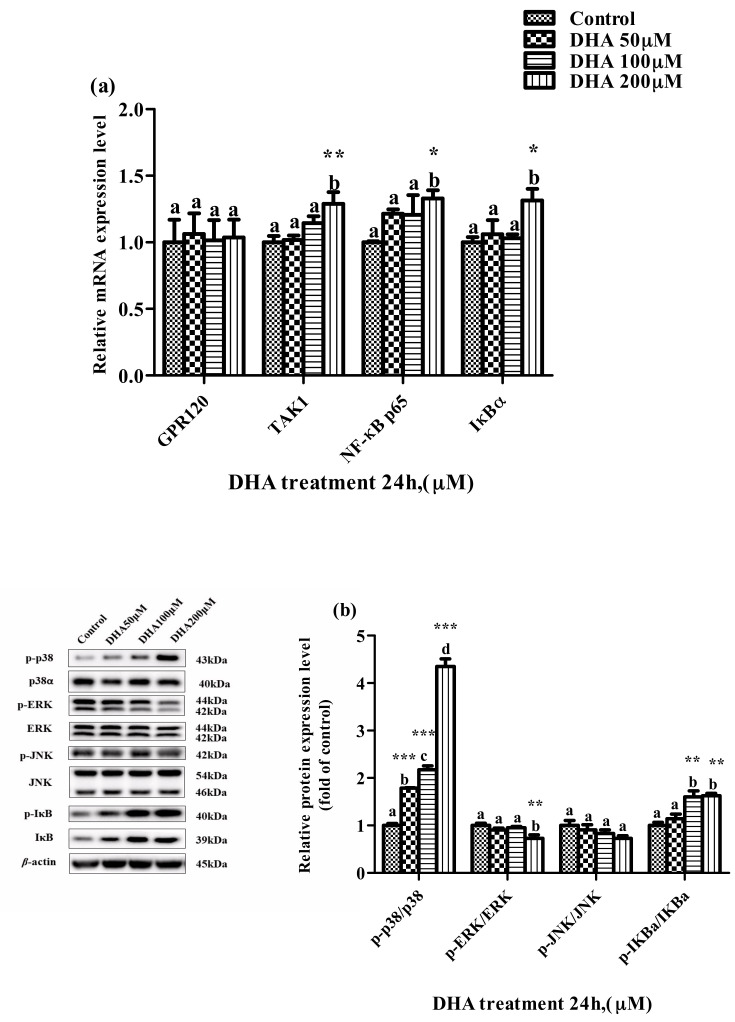
The effects of DHA-treated BRL-3A for 24 h on the expression of the target genes mRNA (**a**) and proteins (**b**) related to MAPK and NF-κB pathways. Means with different letters are significantly different from one another (*p* < 0.05) as determined by variance analysis followed by multiple comparisons with LSD post-hoc test; * statistically significant differences vs. control (*p* < 0.05); ** statistically significant differences vs. control (*p* < 0.01); *** statistically significant differences vs. control (*p* < 0.001).

The relative expression levels of TAK1, NF-κB p65 and IκBα mRNA were higher in the 200 μM DHA treatment than those in the control. The ratio of p-IκBα/IκBα was significantly increased in the 50, 100 and 200 μM DHA treatments, and the ratio of p-p38/p38 was increased parallel to the increase in DHA from 50 μM to 200 μM (*p* < 0.05) (Figure 6a,b). However, the ratio of p-ERK/ERK protein in the BRL-3A cells from the 200 μM DHA treatment was lower than that from the control and other treatment groups (*p* < 0.05).

## 4. Discussion

In this study, DHA treatment on rat liver BRL-3A cells for 24 h promotes cell apoptosis and inflammatory response, and the p38 MAPK, ERK and NF-κB signal pathways were involved in mediating the apoptosis and inflammatory response. DHA has cytotoxicity to various types of cancer cells (e.g., lung cancer cell), and the cytotoxicity of DHA affected by both the dose and the treatment time [17,18,19]. So far, the reports about the cytotoxicity of DHA on normal cells are limited. Zajdel et al. (2010) reported that the DHA pre-treatment increased the sensitivity of non-cancer cells to drug toxicity [20]. In addition, the previous study reported that the cell activity of rat pancreatic β cells (RINm5f) decreased after 100 μM DHA treatment for 24 h [21].

LDH is recognized as a marker for the cell membrane injury, cell death and liver injury [22]. In this study, the release of LDH increased, but the cell viability decreased with the increase in DHA concentration from 150 μM to 300 μM DHA in a dose-dependent manner, and the proportion of apoptotic cells was increased in all the DHA treatment groups. These results suggested that DHA may cause injury to BRL-3A cells.

The levels of AST and ALT are widely recognized to be the biomarkers of liver function or liver injury [23]. AKP is another of the important biomarkers of the damage to liver cells, and usually provides diagnostic information for liver function or chronic liver disease [24]. The increased secretion of AKP by liver cells is a sign of cholestatic hepatitis, and AKP is highly expressed in patients with various cancers [25,26]. In our study, DHA might cause injury to BRL-3A cells according to the change in the levels of ALT, AST and AKP in the supernatant after different concentrations of DHA treatment. The results of flow cytometry and fluorescence microscopy further confirmed that the treatment of DHA on BRL-3A cells increases the cell apoptosis.

The levels of TG, T-CHO, LDL-C and HDL-C in the liver were used to evaluate the protective effect of the liver. DHA can reduce the TG concentration in the liver, increase the level of HDL-C in the liver and protect the liver from fatty liver, but the inconsistent studies show that the treatment of DHA would increase liver fat and increase the susceptibility of the liver to pathogen stimulation [27,28,29]. The cytoplasmic space of hepatocytes was occupied by excessive lipids, which might affect the function of the hepatocytes and make the hepatocytes vulnerable to lipopolysaccharides (LPS) [30]. In our study, the increase in the TG, T-CHO and HDL-C in BRL-3A cells from the 200 μM DHA treatment indicated that the treatment of 200 μM DHA might affect the protective ability of the BRL-3A cells.

Inflammatory cytokines play a major role during the process of liver inflammation, especially from steatosis to steatohepatitis, or from inflammatory infiltration to liver injury [31]. Previous studies reported that carbohydrates, therapeutic drugs and the hypoxia/reoxygenation model could increase the levels of IL-1 β and IL-6 in the supernatant of BRL-3A cells [13,32,33]. Inflammation interferes with the expression of genes related to lipid metabolism, leading to lipid accumulation [32]. In our study, the level of IL-6 in the supernatant increased in all of the DHA treatment groups. As mentioned above, the treatment of DHA may cause an inflammatory response in the BRL-3A cell.

The NF-κB signal pathway is activated by the p-IκBα protein, which makes it enter from the cytoplasm to the nucleus and enhance the production of pro-inflammatory cytokines, such as IL-1β and IL-6. The MAPK signal pathway family mainly includes the ERK, JNK and p38 pathways [34,35]. The activation of p38 MAPK can promote the secretion of IL-6 and activate the NF-κB pathway [10]. ERK1/2 participate in the regulation of cell survival, and the continuous activation of ERK is conducive to cell survival [36,37]. The activation of ERK1/2 can inhibit the activity of NF-κB in hepatocytes, thus regulating the inflammatory response of hepatocytes and facilitating their survival [30]. In addition, continuous ERK activation can promote the production of IL-10, which can inhibit NF-κB from entering the nucleus, thus alleviating the inflammatory response [38]. In our study, the level of IL-10 was decreased in the 200 μM DHA treatment, and the relative expression of TAK1, NF-κB p65 and IκBα mRNA was increased in the 200 μM DHA treatment; the ratio of p-IκBα/IκBα was increased in the 50, 100 and 200 μM DHA treatments, and the ratio of p-p38/p38 was increased with the increase in DHA concentration from 50 to 200 μM. In addition, the ratio of the p-ERK/ERK protein was decreased in the 200 μM DHA treatment. These results indicate that DHA exhibits cytotoxicity by activating p38 MAPK and NF-κB signal pathways and induces cell death by inhibiting the ERK activation signal pathway. Further studies need to prove the mechanism of DHA cytotoxicity and direct the clinic use of DHA for human health.

## 5. Conclusions

**Figure 7:**
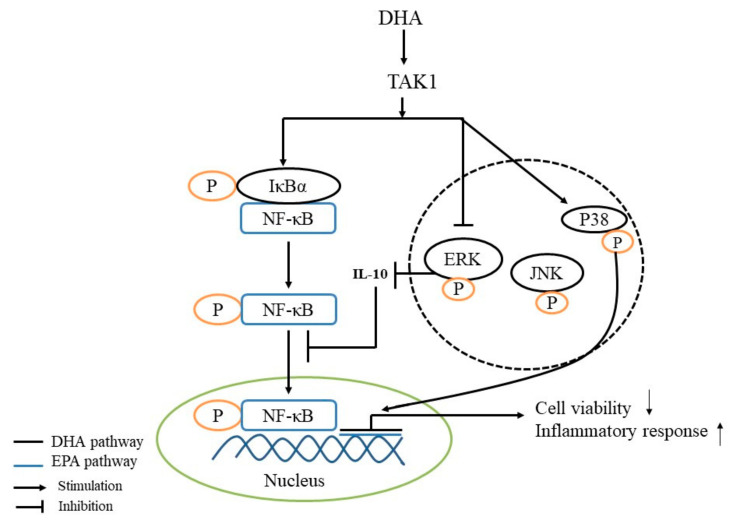
Schematic mechanisms illustrating the DHA-treated BRL-3A for 24 h on the mechanism of inflammatory response regulation.

In conclusion, DHA-treated (50, 100 and 200 μM) rat liver BRL-3A cells for 24 h promote cell apoptosis and the inflammatory response, and the p38 MAPK, ERK and NF-κB signal pathways are involved in mediating the apoptosis and inflammatory response (Figure 7).

## Figures and Tables

**Table 1:** Primers used for all target genes.

Target Genes	Sequence 5′-3′	Reference
β-actin	F:5′-AGTGTGACGTTGACATCCGTAA-3′	[12]
	R:5′-GGACAGTGAGGCCAGGATAGA-3′	
IκBα	F:5′-TGAAGTGTGGGGCTGATGTC-3′	[13]
	R: 5′-AGGGCAACTCATCTTCCGTG-3′	
NFκBP65	F: 5′-CATACGCTGACCCTAGCCTG-3′	[13]
	R: 5′-TTTCTTCAATCCGGTGGCGA-3′	
TAK1	F:5′-TCTGGATGTCCCTGAGATCGT-3′	[14]
	R:5′-GCTCACCTGACCAGGTTCTG-3′	
GPR120	F:5′-GCATAGGAGAAATCTCATGG-3′	[15]
	R:5′-GAGTTGGCAAACGTGAAGGC-3′	

TAK1 = transforming growth factor-β-activated kinase 1, GPR120 G = protein coupled receptor 120, NF-κB p65 = nuclear factor-B p65, IκBα = inhibitor of NF-κB.

## Data Availability

All data presented to support the findings of our study are included in the manuscript.
